# Mitochondrial Genetic Background Shapes Baseline Retinal Function, Age‐Related Bioenergetic Remodeling, and Microglial STING Phenotypes

**DOI:** 10.1111/acel.70771

**Published:** 2026-10-15

**Authors:** McKenna M. Somerville, Sydney S. Lin, Scott W. Ballinger, Alecia K. Gross

**Affiliations:** ^1^ Department of Neurobiology, Heersink School of Medicine University of Alabama at Birmingham Birmingham Alabama USA; ^2^ Department of Pathology, Heersink School of Medicine University of Alabama at Birmingham Birmingham Alabama USA

**Keywords:** metabolism, microglia, neuroinflammation, retina

## Abstract

Mitochondria are central regulators of retinal bioenergetics and innate immune signaling, yet how mitochondrial genetic background and aging jointly influence immunometabolic responses in the retina remains poorly defined. Here, we investigated the effects of mitochondrial DNA (mtDNA) variation on retinal function and the combined effects of mtDNA variation and age on bioenergetic composition, mtDNA damage, and the microglial expression and intracellular localization of stimulator of interferon genes (STING) protein. Using mitochondrial–nuclear eXchange (MNX) mice harboring identical C57BL/6J nuclear genomes but distinct C57 or C3H mtDNA haplotypes, we assessed oxidative phosphorylation complex composition, electrophysiological function, mtDNA damage, and microglial STING dynamics across the lifespan and following acute intraocular pressure (IOP) elevation. Aging was associated with increased mtDNA damage and remodeling of oxidative phosphorylation complex composition, characterized by reduced Complex III and V abundance and increased Complex IV. Despite these age‐associated changes, photoreceptor‐ and retinal ganglion cell‐associated retinal responses were preserved at early ages, whereas bipolar cell‐associated responses differed by mtDNA background. Microglial STING expression increased with age. Acute IOP elevation was associated with differential microglial STING dynamics and STING association with lysosomal compartments decreased with age. Together, these findings demonstrate that mitochondrial genetic background and aging influence retinal bioenergetics and neuroimmune protein expression and identify mtDNA variation as a modifier of age‐associated microglial STING expression in the retina.

## Graphical abstract

Mitochondrial Genetic Background Shapes Neuroimmune Aging and Retinal Bioenergetic Remodeling, Authors: McKenna M. Somerville, Sydney S. Lin, Scott W. Ballinger, Alecia K. Gross. This paper uses a mitochondrial‐nuclear exchange mouse model to explore the impact of mitochondrial DNA variation and age on retinal immunometabolism, bioenergetics, and function.

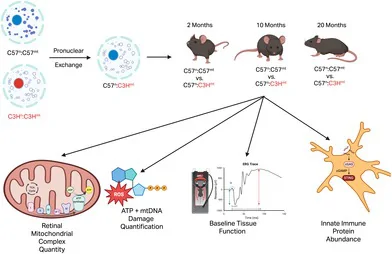

## 1 Introduction

Mitochondria play a critical role in regulating retinal physiology and function, as the retina has one of the highest metabolic demands of any tissue in the body (Country 2017; Linsenmeier and Braun 1992; Liu and Prokosch 2021; Winkler 1981; Wong‐Riley 2010). Beyond their canonical role in ATP production, mitochondria act as signaling hubs that integrate bioenergetics, redox balance, and innate immune activation. Disruption of mitochondrial homeostasis, through aging, genetic variation, or stress, can lead to the release of mitochondrial components, including mitochondrial DNA (mtDNA), that serve as damage‐associated molecular patterns (DAMPs) capable of activating innate immune pathways. Among these pathways, the cyclic GMP‐AMP synthase—stimulator of interferon Genes (cGAS‐STING) has emerged as a central mediator of inflammation. In response to cytosolic mtDNA, cGAS‐STING triggers the release of proinflammatory cytokines, further exacerbating inflammation (Dvorkin et al. 2024).

cGAS‐STING expression in microglia, the resident immune cells of the central nervous system and retina, is increasingly recognized as a key contributor to neuroinflammation across aging and disease (Gulen et al. 2023). Likewise, increased STING protein abundance has been documented in aged brains (Passarella et al. 2024), and altered microglial STING signaling has been implicated in neurodegenerative disorders, including amyotrophic lateral sclerosis, Parkinson's disease, Alzheimer's disease, traumatic brain injury, and glaucoma (Gulen et al. 2023; Liu et al. 2024; Paul et al. 2021). Aging alters mitochondrial homeostasis and immunometabolic signaling, influencing disease susceptibility and retinal response to stress, giving rise to a chronic, low‐grade inflammatory state termed “inflammaging” (Tamatta et al. 2025). Given the established relationship between mitochondrial stress, cGAS‐STING signaling, and neuroinflammation, age‐associated changes in STING abundance and intracellular localization may provide insight into how mitochondrial genetic variation influences retinal neuroimmune phenotypes. Whether inherited mitochondrial genetic variation contributes to age‐dependent differences in microglial STING abundance and intracellular localization, particularly in the aging retina, remains poorly defined.

MtDNA exhibits extensive natural genetic diversity across populations, and even modest mtDNA sequence differences can significantly alter oxidative phosphorylation efficiency, reactive oxygen species (ROS) production, and susceptibility to cellular stress (Kobayashi and Imanaka 2025). Experimental models using mitochondrial–nuclear eXchange (MNX) mice have demonstrated that mtDNA background can dictate bioenergetic capacity and disease vulnerability in peripheral tissues, including the heart, lung (Kandasamy et al. 2019), and liver (Kesterson et al. 2016). However, whether mitochondrial genetic background shapes age‐dependent differences in mitochondrial composition, retinal function, and microglial STING expression and STING localization has not yet been explored. This represents an important gap, as mitochondrial–nuclear interactions and mtDNA‐dependent differences in immunometabolic responses may contribute to variation in susceptibility to age‐related retinal disease.

The retina provides an accessible tissue to study these interactions, combining high mitochondrial demand, vulnerability to oxidative and mtDNA damage, tightly regulated neuron‐glial interactions, with a well‐established functional readout that sensitively reflects neuronal function, which is altered by mitochondrial dysfunction (Mey and DeSilva 2023; Schon and Manfredi 2003). Retinal microglia play a pivotal role in immune surveillance and inflammatory responses, making the retina an ideal tissue to examine how mitochondrial genetics and aging work together to influence microglial STING abundance. Further, stress models, such as transient elevations in intraocular pressure (IOP), provide a controlled way to probe mitochondrial and immune responses to injury‐relevant stimuli.

Here, we used an aged MNX mouse model containing identical C57BL/6J nuclear genomes, but either C57BL/6J or C3H/HeN mtDNA, to examine how mitochondrial genetic background alters retinal function, as well as how aging and mitochondrial genetic background together shape retinal bioenergetics, retinal function, and microglial STING expression. We utilized biochemical, electrophysiological, and immunohistochemical studies across the lifespan and following acute IOP stress to define how inherited mtDNA influences innate immune signaling in the aging retina (Figure 1). These studies identify mitochondrial genetic background as a modifier of age‐associated differences in microglial STING abundance and intracellular localization, providing evidence that inherited mtDNA variation may contribute to differences in retinal immunometabolic homeostasis.

**FIGURE 1:**
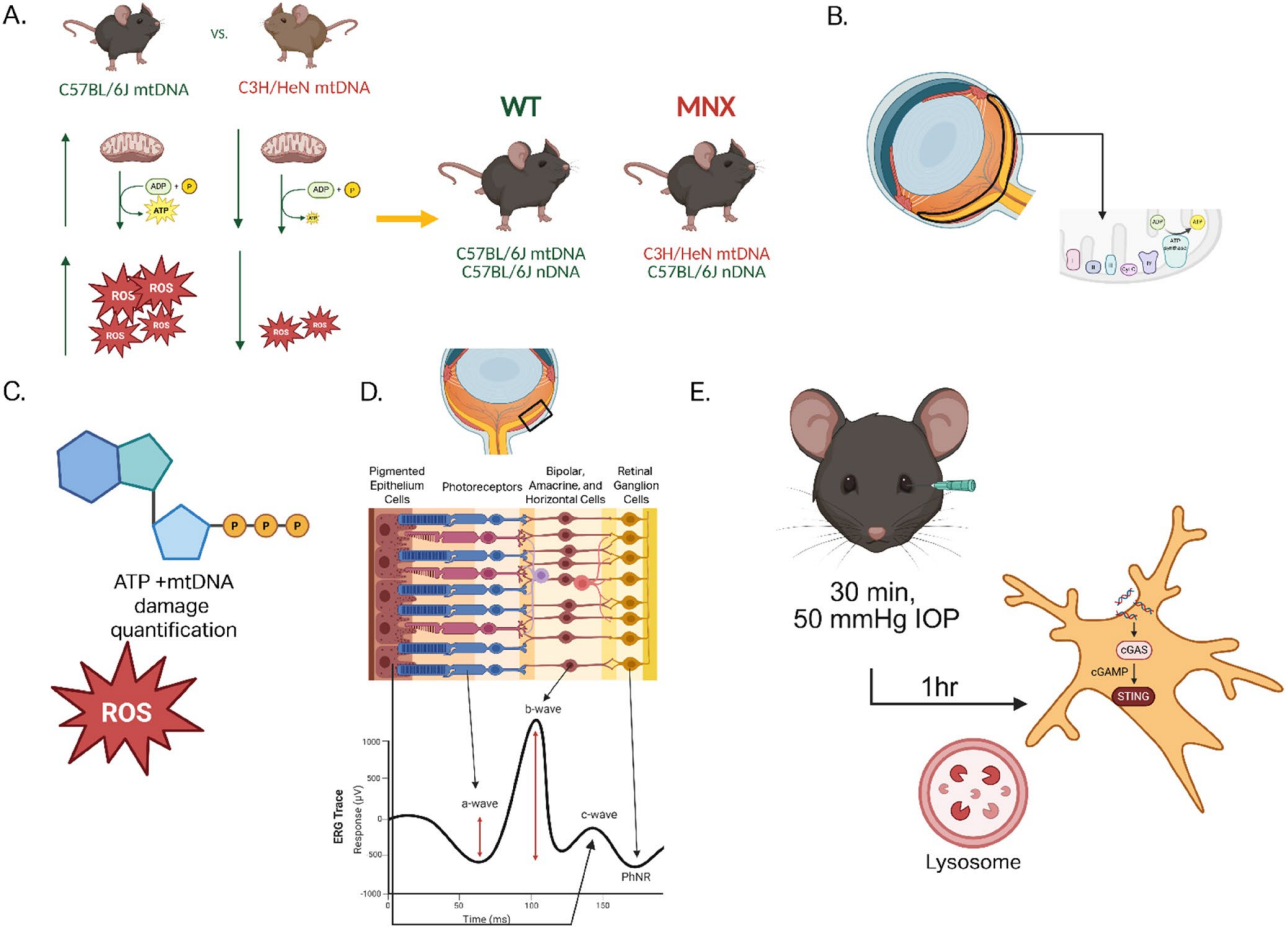
Schematic diagram of experiments. (A) Illustration of the differential mitochondrial efficiency and reactive oxygen species (ROS) production seen across C57BL/6J and C3H/HeN mice. Pronuclear exchange generates WT mice with both C57BL/6J nuclear and mitochondrial genomes and MNX mice with C57BL/6J nuclear and C3H/HeN mitochondrial genomes. (B) Retina harvested and oxidative phosphorylation composition assessed. (C) ROS and ATP quantity measurement. (D) Retinal function evaluated via electroretinography. (E) Immunologic response to acute intraocular pressure (IOP) increase assessed. Schematic generated using Biorender.

## 2 Materials and Methods

### 2.1 Research Animals

Mitochondrial Nuclear eXchange (MNX) mice were obtained from colonies established at the University of Alabama at Birmingham, have been previously described, and are genetically validated in lab using methods outlined here (Kesterson et al. 2016). Briefly these mice share the same nuclear background (C57BL/6J) but have distinct mtDNA backgrounds (C57BL/6J or C3H/HeN). For this study, male and female C57BL/6J mice harboring either a C57BL/6J or C3H/HeN mtDNA (C57^n^:C57^mt^ or C57^n^:C3H^mt^, respectively), totaling approximately 113 mice, were used. Mice were maintained under 12‐h cyclic dark/light conditions in standard cages and fed ad libitum. Mice were aged in this environment to approximately 2 months, 10 months, and 20 months of age. All animal studies were conducted in compliance with the National Institutes of Health Guide for the Care and Use of Laboratory Animals and were approved by the Institutional Animal Care and Use Committee of the University of Alabama at Birmingham. Experimental cohorts were distributed across assays as follows: Western blot and mtDNA damage analyses were performed on mice at 2, 10, and 20 months of age (*n* = 4 per genotype per timepoint, except for 10‐month mtDNA damage experiments, which had *n* = 3). Immunohistochemistry and acute IOP experiments included mice from all age and genotype groups (*n* = 3 per group for naïve immunohistochemistry and *n* = 5 per group for IOP experiments and subsequent immunohistochemistry). ERG and ATP analyses were performed in younger cohorts (3 weeks to 2 months) due to study design constraints.

### 2.2 Electroretinograms (ERGs)

ERGs were performed on 3‐week‐old (*n* = 7, 11 M/4F) and 2‐month‐old (*n* = 5, 6 M/4F) C57BL/6 wild‐type and C57^n^:C3H^mt^ mice using the Celeris D430 rodent ERG system (Diagnosys LLC, Lowell, MA, USA) equipped with 3‐mm standard luminance stimulators and integrated light‐guide electrodes. Mice were dark‐adapted overnight prior to recordings, and experiments were performed under dim red light. Anesthesia was induced by intraperitoneal injection of ketamine (71.5 μg/g) and xylazine (14.3 μg/g) prepared in 0.1 M phosphate‐buffered saline (PBS). Pupils were dilated with a 1:1:1 mixture of 0.5% phenylephrine, 0.5% proparacaine hydrochloride, and 1% tropicamide. The corneal surface was covered with Sentrx Eye Lube Pro, and the stimulators were positioned in direct contact with the cornea. Diagnosys touch‐touch protocols were used to record ERGs from one eye at a time, with the contralateral eye serving as reference. Scotopic ERGs were recorded using 1.0‐Hz flashes of white light (6500 K) at the following flash intensities: 0.01, 0.1, and 1.0 cd·s/m^2^. For each stimulus intensity, three responses were recorded and averaged. A‐wave amplitudes were measured from baseline to the negative trough, and b‐wave amplitudes were measured from the a‐wave trough to the major cornea‐positive peak. To elicit the c‐wave, a 1.0‐Hz flash of white light (6500 K) at 150 cd/m^2^ was administered, and the c‐wave amplitude was measured from baseline to the peak following the b‐wave trough. Mice were then light adapted for 10 min with a 20 cd/m^2^ green background light. Under photopic conditions, ERGs were recorded using 2.0‐Hz flashes of white light (6500 K) at 20 cd·s/m^2^. The photopic negative response (PhNR) was measured from baseline to the negative trough following the b‐wave, reflecting cone‐mediated RGC responses (Chrysostomou and Crowston 2013; Maloney et al. 2020). Implicit times (latencies) were defined as the time from flash onset to the peak (or trough) of the respective waveform component and are reported in milliseconds. PhNR intensities were normalized via corresponding *b*‐wave.

### 2.3 Pan—OXPHOS Western Blotting

Pan‐OXPHOS western blots were performed on retinal tissue from male and female C57^n^:C3H^mt^ (MNX) and C57^n^:C57^mt^ (WT) mice at 2, 10, and 20 months of age. Two retinas (1 mouse) were harvested and pooled from male and female mice per genotype and age group (*n* = 4) and homogenized using a mitochondria‐optimized RIPA buffer (50 mM Tris–HCl, pH 7.4, 150 mM NaCl, 1% NP‐40 mL, 0.25% sodium deoxycholate). Retina from each eye was harvested and pooled to serve as a single biological replicate. Protein concentration was determined using Pierce BCA kit (Thermo Scientific 23,225). 10 μg of protein was mixed with 4× Laemmli buffer (Thermo Scientific, J60015.AD) and deionized water. The mixture was incubated at 50°C for 5 min. Protein in technical triplicate was run through a 4%–20% gradient SDS‐PAGE gel (BIO‐RAD, 4561096). The gel was run at 100 V until the dye front reached the bottom of the gel. Subsequently, a wet transfer was performed using activated 0.45 μm PVDF membrane (Thermo Scientific, 88,518). Transfer was performed at 100 V for 1 h at 4°C. After transfer, the membrane was blocked in 5% BSA in TBS‐T for 1 h at room temperature with gentle rocking. Subsequently, membrane was incubated with Total OXPHOS Rodent WB Antibody Cocktail (Abcam, ab110413) diluted 1:1000 in 5% BSA/TBS‐T and beta actin (Rabbit) (Abcam, ab8227) 1:1000 diluted 1:1000 in 5% BSA/TBS‐T overnight at 4°C. For secondary antibody incubation, membranes were washed 3 times with TBS‐T for 5 min each wash. Membranes were subsequently incubated with anti‐mouse (Licor, 926‐68070) and anti‐rabbit (Licor, 926‐32211) secondary antibodies diluted 1:5000 in 5% BSA/TBS‐T for 1 h at RT. Following an additional 3 TBS‐T 5 min washes, membranes were imaged using licor odyssey imaging system. The bands were quantified using ImageJ, with beta actin used as a loading control.

### 2.4 Immunohistochemistry

Enucleated eyes from male and female mice from both genotype groups across all age groups, with and without IOP treatment, were placed in 4% paraformaldehyde in 0.1 M PBS (pH 7.4) at room temperature for 30 min, then incubated in 30% sucrose in PBS at 4°C for at least 1 h. Eyes were embedded in Tissue‐Tek O.C.T. (Sakura Finetek, Torrance, CA, USA), flash frozen in ethanol cooled to near freezing by dry ice, and sectioned into 10‐μm‐thick slices using a cryomicrotome. Following sectioning, the eyes were rinsed in PBS. For antibodies that require it, heat‐mediated sodium citrate buffer antigen retrieval was performed for 15 min at 65°C. Slides were subsequently allowed to cool for 15 min to help with tissue‐slide adhesion. Slides were rinsed with PBS, then incubated for 1 h at room temperature in a blocking buffer: 2.5 g BSA, 2.5 mL goat serum, 50 mL PBS, 50 μL Tween‐20 (0.1%), 150 μL Triton X‐100 (0.3%). Following blocking, slides were incubated in primary antibody overnight at 4°C at the following concentrations in blocking buffer solution listed above: STING (CS, 13647 T) (1:300), IBA1‐647 (Abcam ab225261) (1:500), LAMP1‐647 (Abcam, ab237307) (1:400). After 3 rinses with PBS, the slides were incubated in secondary antibody for 1 h at room temperature at the following concentrations in Goat anti‐rabbit 555 (Thermo A21428) (1:1000). DAPI was diluted 1:10,000 in PBS and incubated for 15 min at room temperature. Following an additional 3 rinses with PBS, slides were mounted with mounting medium (Invitrogen P36930) and cover slipped. Images were taken in technical triplicate at 40X in oil immersion on a Nikon AX‐R fluorescence confocal microscope. Images were analyzed using Imaris software for 3D analysis using surface creation. Imaris software was used to quantify STING, IBA1, and LAMP1 signal, as well as their colocalization and spatial relationships. Images were analyzed in a blinded manner using standardized analysis parameters that were batch‐applied across the image dataset to ensure consistent analysis. In microglia IBA1/STING dataset, total number of STING inside of IBA1 was normalized to number of microglia per image to account for inter‐image variability in microglia counts.

### 2.5 Acute IOP Elevation

Acute IOP elevation was induced from male and female mice from both genotype groups across all three age groups (*n* = 5, 2 month WT: 2 M/3F, 2 month MNX: 3 M/2F, 10 month WT: 3 M/2F, 10 month MNX: 2 M/3F, 20 month WT: 2 M/3F, 20 month MNX: 2 M/3F) according to the syringe pump methods previously described (Crowston et al. 2015), with the following deviations: the use of isoflurane for anesthetic in place of a ketamine/xylazine mixture, and a 30G needle was used in place of pulled glass (BD, 305106). After 30 min, 50 mmHg IOP elevation described above, mice were returned to baseline IOP and allowed to recover for 1 h before euthanasia via cervical dislocation and decapitation. After IOP elevation, eyes were fixed and processed for immunohistochemistry as described above (2.4).

### 2.6 ATP Assay

Acid extraction was performed on 2 flash frozen retinas per mouse from non‐IOP male and female mice of both genotypes at 8 weeks of age (*n* = 8) to retrieve ATP as follows: 160 μL ice‐cold 0.5 M perchloric acid (PCA) was added to retina. On ice, retinal samples were homogenized using a plastic pestle (Grainger, 6HAY3) for approximately 30 s per sample. Homogenates were incubated on ice for 10 min. Following incubation, sample was centrifuged at 16,000 g for 10 min at 4°C. The supernatant was recovered, avoiding protein pellet. To neutralize PCA, titrations were performed using 2 M KOH and 0.2 M KOH. Once pH of 6–7 was achieved, 40 μL of 0.3 M MOPS, pH 7.0 was added. After an additional 5‐min incubation on ice, an additional centrifugation was performed at 15,000 × g for 5 min at 4°C. ATP quantification was performed using a luciferase assay kit (Thermo Fisher A22066) and the plate was read using a Varioskan LUX Multimode Microplate Reader.

### 2.7 mtDNA Damage Assay

MtDNA damage was assessed in whole retinal tissue using a quantitative assay that compares amplification of short and long mtDNA fragments (Yakes and Van Houten 1997). This assay was performed as previously described (Ballinger et al. 1999) on male and female mice from each genotype group across all three age groups (*n* = 3) with minor modifications including the use of GoTaq Mastermix (Promega M7122) for the short sequence reaction and Phusion Polymerase Plus (Thermo Fisher F632S) and 1 uL of 100% DMSO for the long sequence. Band intensities were normalized to loaded PCR volume prior to calculation of long: short mtDNA ratios. The damage index to quantify mtDNA damage was calculated as the ratio of long to short mtDNA amplicon abundance (long DNA PCR product/short DNA PCR product). Data were log‐transformed prior to analysis to improve compliance with model assumptions. MtDNA lesion calculations were performed using 2‐month‐old C57n:C57mt tissues as control; equation – ln (mtDNA damage ratio/control).

### 2.8 Statistical Analysis

All statistical analyses were performed using GraphPad Prism (v11.0.2) and RStudio. ERG data were exported and initially processed in Microsoft Excel prior to analysis. Three‐way repeated‐measures ANOVA was used to evaluate the effects of age, stimulus intensity, and mtDNA background. Where appropriate, unpaired *t*‐tests with Welch's correction were performed. Western blot band intensities were averaged across technical replicates and analyzed for each respiratory complex using two‐way ANOVA followed by Tukey's multiple‐comparisons test. Confidence intervals (CI) were calculated at the 95% level (Supporting Information). Due to concerns of β‐actin loading control reliability, an additional regression analysis was performed with OXPHOS expression as the dependent variable and age, genotype, and β‐actin intensity as predictors. Age remained a highly significant predictor of OXPHOS expression (F (2,19) = 38.96, *p* = 1.89 × 10^−7^), whereas β‐actin expression was not significantly associated with OXPHOS levels (F (1,19) = 0.67, *p* = 0.422). When OXPHOS normalized to β‐actin levels were assessed, age remained a significant predictor of normalized OXPHOS expression (ANOVA: F (2,18) = 9.59, *p* = 0.0015).

Immunohistochemistry datasets obtained under naïve conditions were analyzed using two‐way ANOVA with age and mtDNA background as fixed factors, followed by Šídák's multiple‐comparisons testing. Acute IOP immunohistochemistry datasets were analyzed using linear mixed‐effects models in R, with age, mtDNA background, and treatment (control or 50 mmHg IOP) included as fixed effects and MouseID included as a random effect to account for paired measurements obtained from contralateral eyes of the same animal. Post hoc comparisons were performed using estimated marginal means (emmeans) with Tukey adjustment to evaluate treatment effects within age and genotype groups, as well as genotype effects within age and treatment groups.

mtDNA damage data were analyzed using two‐way ANOVA followed by Šídák's multiple‐comparisons test. Outliers were assessed using Grubbs' test. One potential outlier was identified at the 20‐month timepoint. Sensitivity analyses indicated that exclusion of this observation eliminated statistical significance; however, no technical or experimental basis for exclusion was identified. Therefore, all observations were retained for the final analyses.

For all studies, sample sizes of 3–4 animals per group were used because the study relied on locally aged, non‐commercial mitochondrial‐nuclear exchange (MNX) mouse colonies, limiting animal availability. These exploratory sample sizes may reduce statistical power, particularly for interaction analyses, and findings should be interpreted accordingly. Further, formal sex‐covariate modeling was performed only for the acute IOP dataset. For all other experiments, sex was assessed qualitatively rather than through formal statistical modeling due to insufficient sample sizes necessary to support sex‐stratified analyses.

## 3 Results

### 3.1 mtDNA and Age Confer Changes in Retinal OXPHOS Composition

Retinal complex I levels, as measured by antibody binding to NADH: ubiquinone oxidoreductase subunit B8, NDUFB8, remained stable with age in C57^n^:C57^mt^ mice (Figure 2B). In contrast, C57^n^:C3H^mt^ mice exhibited increased NDUFB8 levels between 2 and 10 months (*p* = 0.0107, CI = −0.9003 to −0.1143), which were maintained at 20 months of age (*p* = 0.0187, CI = −0.8597 to −0.07372). One data point at the 20‐month timepoint was identified as an outlier by Grubbs' test; however, all data were retained in the analysis. Similarly, complex II (Figure 2C) levels (antibody binding to succinate dehydrogenase subunit 8, SDH8) were unchanged in C57^n^:C57^mt^ mice but increased progressively across all timepoints in C57^n^:C3H^mt^ mice (*p* = 0.0441, < 0.0001, 0.0157).

**FIGURE 2:**
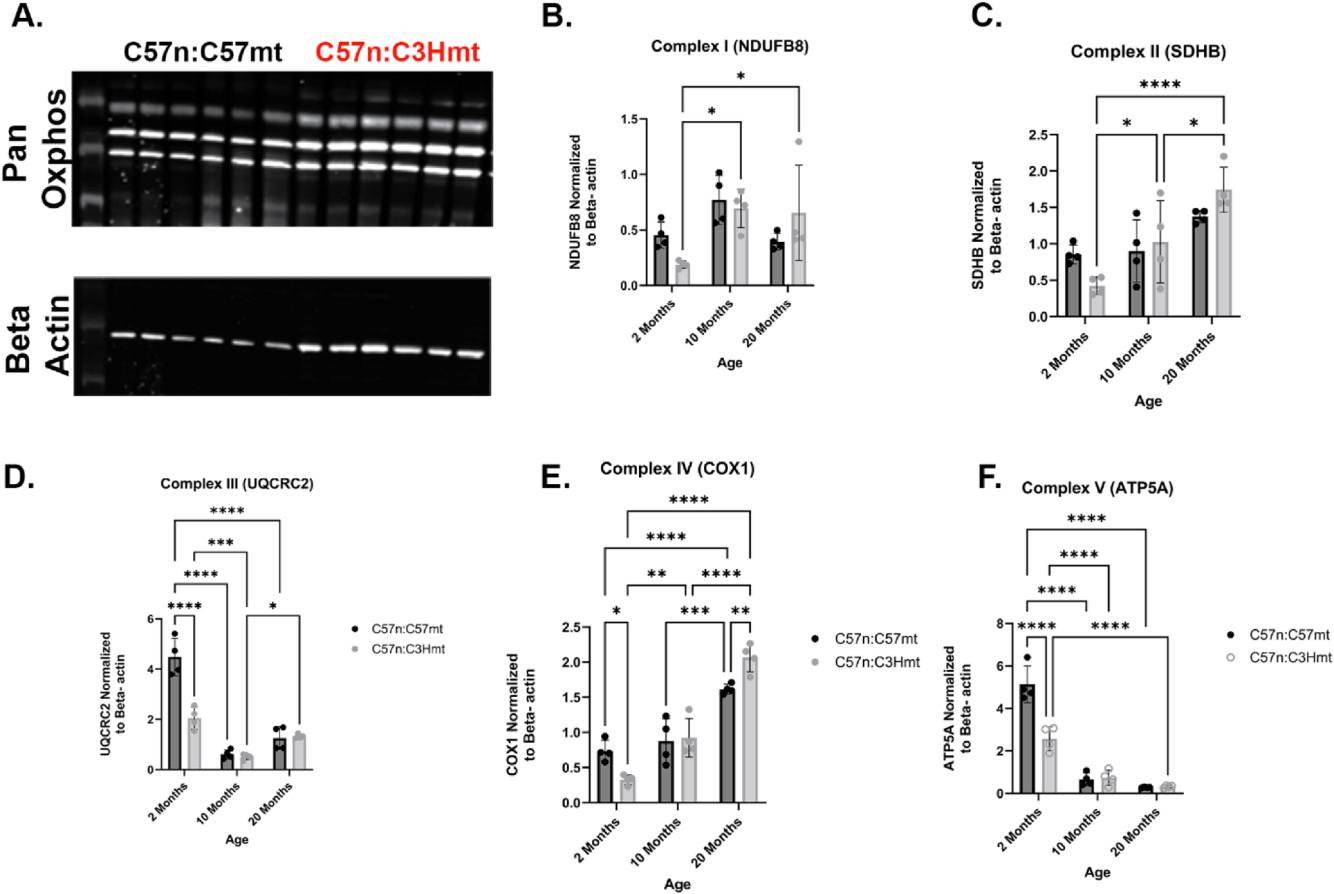
Mitochondrial DNA background and age regulate retinal OXPHOS subunit abundance. (A) Representative image of pan‐oxphos western blotting, done in technical triplicate (*n* = 4) across all three age groups. (B) Quantification of NADH: Ubiquinone oxidoreductase subunit B8 (NDUFB8). Analyzed with two‐way ANOVA, Tukey's post hoc *p* = 0.0107(*), 0.0187(*). (C) Quantification of Succinate Dehydrogenase Complex Iron–Sulfur Subunit B (SDHB). Analyzed with two‐way ANOVA, Tukey's post hoc, *p* = 0.0441(*), < 0.0001(****), 0.0157(*). (D) Quantification of Ubiquinol‐Cytochrome C Reductase Core Protein II (UQCRC2). Analyzed with two‐way ANOVA, Tukey's post hoc, *p* = < 0.0001(****), < 0.0001(****), 0.0001(***), 0.0284(*). (E) Quantification of Cytochrome c oxidase subunit I (COX1). Analyzed with two‐way ANOVA, Tukey's post hoc, *p* = < 0.0001(****), 0.0002(***), 0.0017(**), < 0.0001(****), < 0.0001(****). (F) Quantification of ATP synthase F1 subunit alpha (ATP5A). Analyzed with two‐way ANOVA, Tukey's post hoc, *p* = < 0.0001(****), < 0.0001(****), < 0.0001(****), < 0.0001(****). Dark gray, WT (C57n:C57mt). Light gray, MNX (C57n:C3Hmt). Error bars represent standard deviation.

For complex III, measured by antibody binding to ubiquinol‐cytochrome c reductase core protein 2 (UQCRC2), C57^n^:C3H^mt^ mice showed lower baseline expression at 2 months of age, compared with C57^n^:C57^mt^ mice (*p* = < 0.0001, CI = 1.819 to 3.038) (Figure 2D). Both strains exhibited a marked decline in complex III levels that persisted at 20 months. Complex IV levels (COX1) were also lower in C57^n^:C3H^mt^ mice at baseline (*p* = 0.0113, CI = 0.1048 to 0.7146), but increased with age, surpassing C57^n^:C57^mt^ levels by 20 months (*p* = 0.0061, CI = −0.7553 to −0.1456) (Figure 2E).

Finally, complex V (ATP synthase α‐subunit) expression was initially lower in C57^n^:C3H^mt^ mice compared with C57^n^:C57^mt^ mice (*p* = < 0.0001, CI = 1.872 to 3.252) (Figure 2F). However, unlike complex IV, complex V levels decreased significantly for both strains by 10 months (*p* = < 0.0001, < 0.0001, CI (WT) = 3.636 to 5.312, CI (MNX) = 1.001 to 2.678) and remained reduced at 20 months.

### 3.2 Baseline, Young Retinal Function Differs by mtDNA Background

Retinal function at 2 months, assessed by ERG, was largely preserved in photoreceptors and retinal ganglion cells across mitochondrial backgrounds (Figure 3). Retinal pigmented epithelium firing showed an age‐related difference, with higher amplitudes at 2 months compared to 3 weeks of age regardless of mtDNA background, when analyzed with unpaired *t*‐test with Welch's correction *p* = 0.0041. Inner retinal function, measured by the *b*‐wave amplitude was significantly affected by intensity (*p* < 0.0001), with a significant intensity × genotype interaction (*p* = 0.0385). Neither age (*p* = 0.628) nor genotype (*p* = 0.100) had a significant main effect, and there were no significant interactions between intensity and age (*p* = 0.541), age and genotype (*p* = 0.724), or among age, genotype, and intensity (*p* = 0.907). (Figure 3B).

**FIGURE 3:**
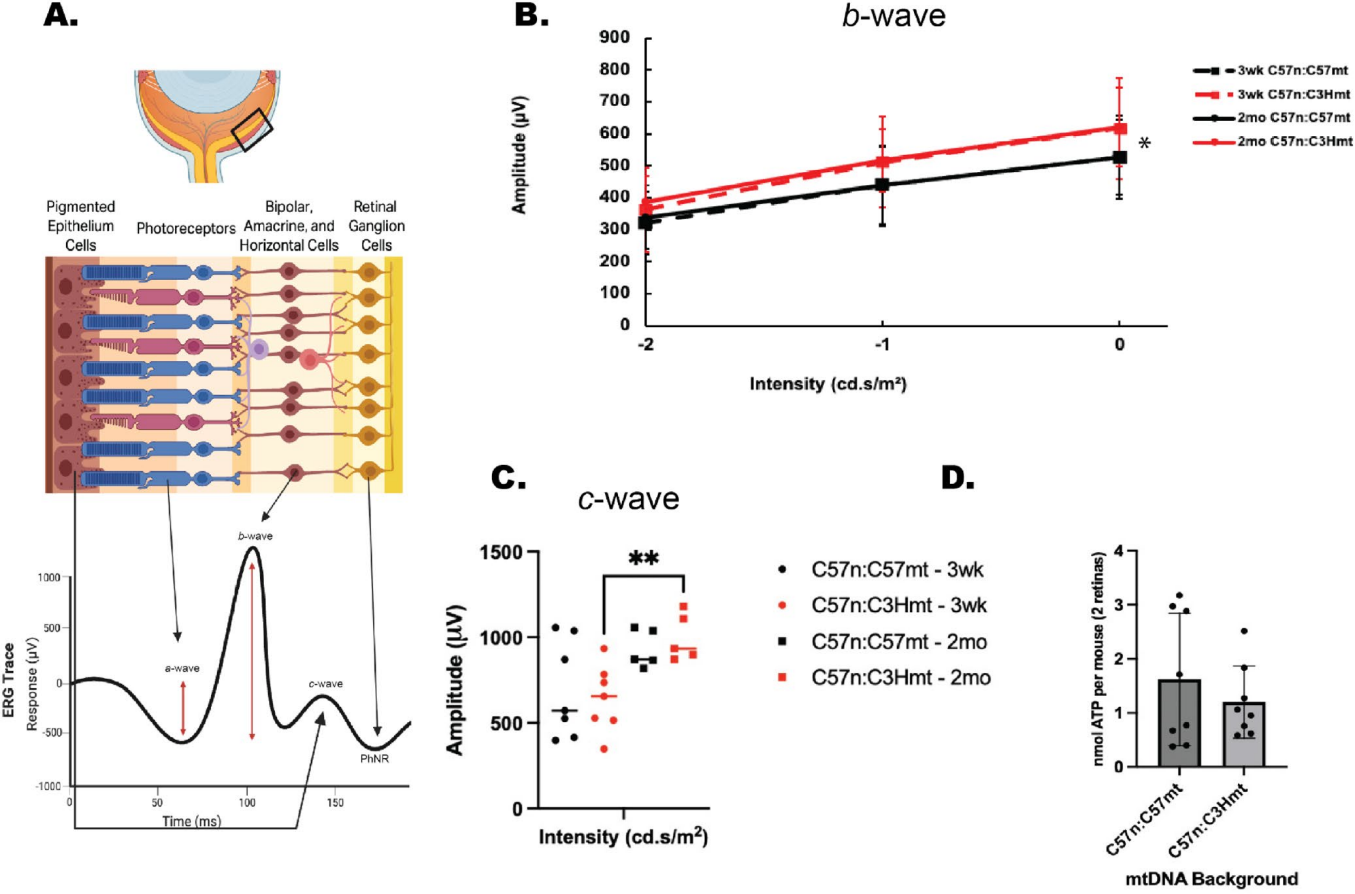
Electroretinograms conducted at 3 and 8 weeks of age reveal functional differences in inner nuclear layer and retinal pigment epithelium (RPE). (A) Schematic depicting Electroretinograms, made in Biorender. (B) *B*‐wave, capturing function of bipolar cells, modulated by Müller glia, analyzed with three‐way repeated measures ANOVA, where Intensity x mtDNA *p* = 0.039(*). (C) *C*‐wave, indicative of RPE function, analyzed with unpaired *t*‐test with Welch's correction *p* = 0.0041(**). (D) Measure of total ATP levels at 8 weeks of age, analyzed with unpaired *t*‐test with Welch's correction, not significant. Error bars represent standard deviation.

At 3 weeks of age, photoreceptors and retinal ganglion cell function remained comparable between groups. Bipolar cell and Müller glia function remained lower in mice of the C57 mtDNA background when compared to mice with C3H mtDNA (Figure 3B). In contrast to the findings at 2 months, RPE function was lower in 3‐week‐old mice of both mtDNA genotypes, with a significant difference between 3 and 8 weeks only in the mice of the C3H background (Figure 3C). ATP levels were not significantly different between mtDNA backgrounds at 2 months of age (Figure 3D).

### 3.3 Age and mtDNA Influence mtDNA Damage

MtDNA damage increased with age in mice with C57 mtDNA (*p* (2 vs. 20 months) = 0.0240, CI = 0.04022 to 0.5889), while there is no significant difference in mtDNA damage with age in mice with C3H mtDNA (Figure 4B). Notably, at 20 months, mice with the C3H mtDNA background exhibited significantly lower mtDNA damage compared with mice with C57 mtDNA (*p* = 0.0404, CI = −0.4278 to −0.01096).

**FIGURE 4:**
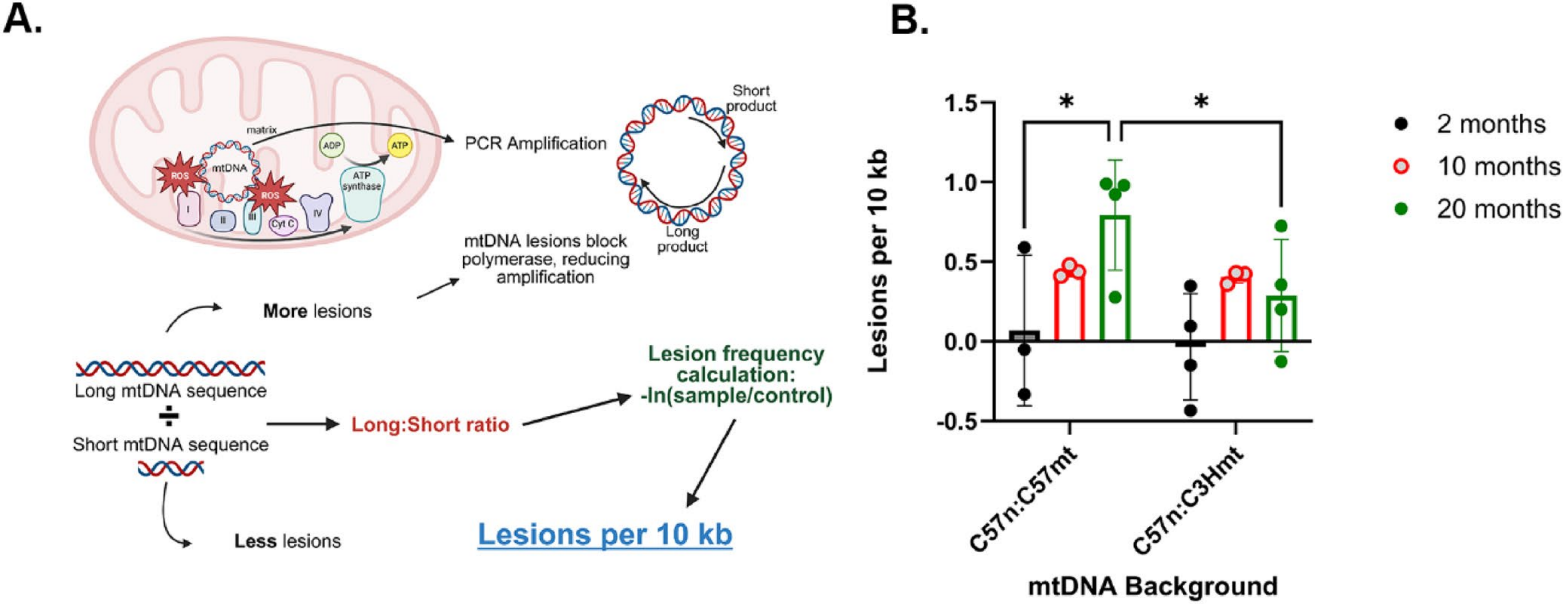
Age and mtDNA influence mtDNA damage. (A) Schematic depicting experiments. PCR reactions were performed for short and long mtDNA amplicons. From this, a mtDNA damage ratio was generated using the relative abundance. A higher ratio is indicative of a decrease in mtDNA damage/lesions, while a lower ratio is indicative of more mtDNA damage/lesions. (B) There is a significant increase in mtDNA lesions as C57n:C57mt animals age, analyzed by Two‐way ANOVA (*p* = 0.024)(*). Tukey's post hoc further revealed that at the 20‐month timepoint, mice with C3H mtDNA background have fewer lesions per 10 kb (*p* = 0.0404)(*). Error bars represent standard deviation.

### 3.4 mtDNA Background and Age Shape Retinal Microglial STING Abundance

Analysis of naïve retinas using IMARIS revealed a significant age‐dependent increase in STING expression within retinal microglia (two‐way ANOVA, age effect: F (2,12) = 21.64, *p* = 0.0001) (Figure 5B,C). Microglial STING expression was elevated in 20‐month‐old animals compared with younger age groups in multiple post hoc comparisons. Neither mtDNA background (*p* = 0.1681) nor the age × mtDNA background interaction (*p* = 0.4433) significantly affected STING expression, though mice with C3H mtDNA had lower microglial STING burden at 20 months when assessed using unpaired *t*‐test (*p* = 0.042). In acute IOP experiments, mixed‐effects modeling accounting for paired eyes revealed a significant main effect of treatment (*p* = 0.0073), age (*p* = 0.0003), and an age × genotype interaction (*p* = 0.0389) on microglial STING abundance. No significant genotype effect, treatment × age interaction, genotype × treatment interaction, or three‐way interaction was observed. (Figure 5E, Supporting Information). Post hoc comparisons demonstrated a significant reduction in STING abundance in 20‐month‐old WT mice following acute IOP elevation relative to contralateral control eyes (estimate: −0.123, CI = −0.233 to −0.0188, *p* = 0.0238), whereas no significant treatment effects were observed in the remaining age and genotype groups. Under contralateral control conditions, which differs from naïve eyes, microglial STING abundance was higher in 2‐month‐old MNX mice than in age‐matched WT mice (estimate: 0.1569, CI = −0.238 to 0.274, *p* = 0.0246). Interestingly however, this directionality flips at older timepoints under control conditions as WT mice exhibited greater microglial STING abundance than MNX mice at 20 months of age at the one‐hour timepoint (estimate: −0.1527, CI = −0.302 to −0.00379, *p* = 0.0455).

**FIGURE 5:**
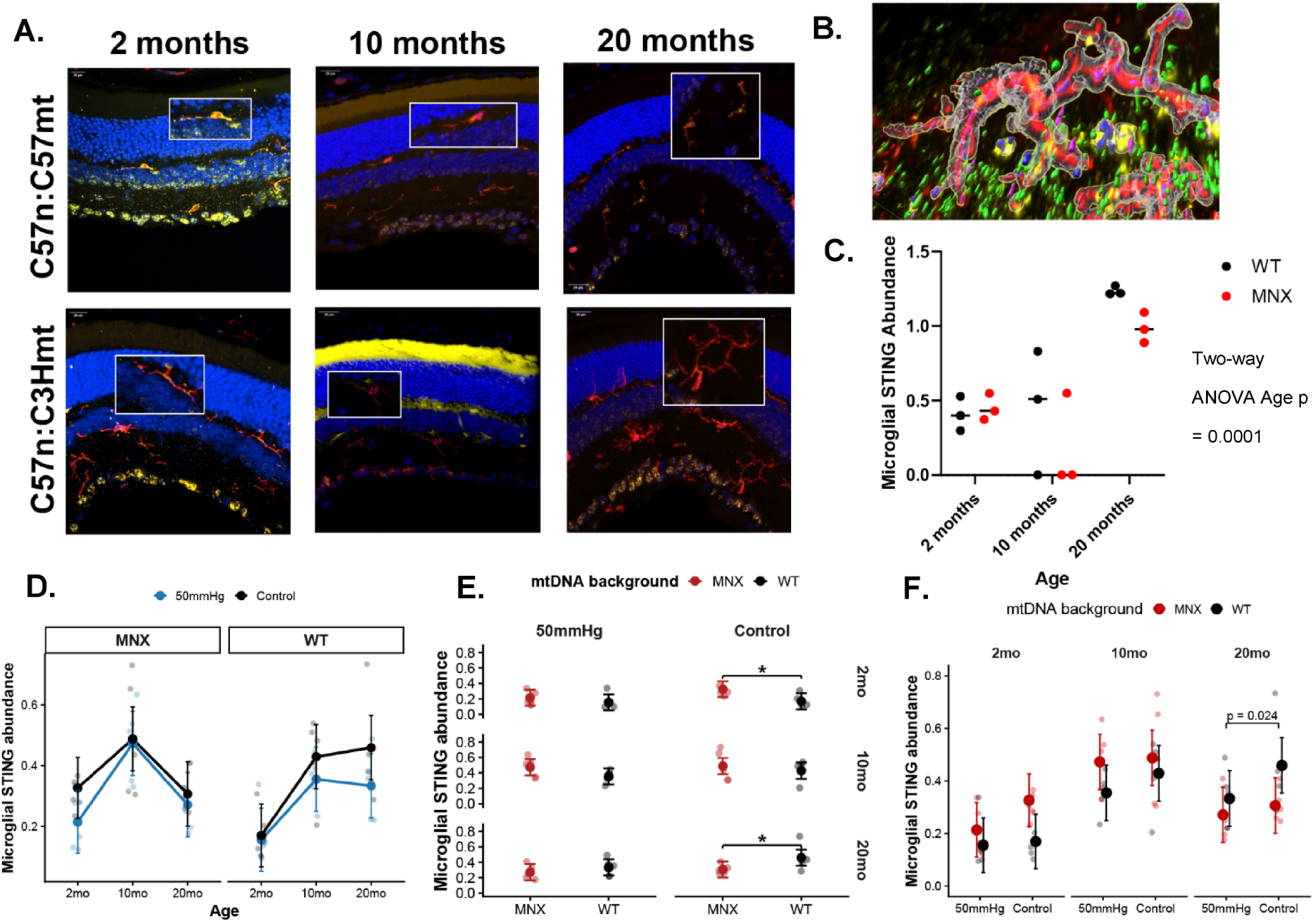
Age, mtDNA background, and acute IOP stress influence microglial STING abundance in the retina. (A) Immunohistochemistry of C57n:C57mt (WT) and C57n: C3Hmt (MNX) retinal slices (Red: IBA1, Yellow: STING scalebar = 20μm) (B) Representative image/schematic of IMARIS surface building and analysis. (C) Age drives Increase in STING in Microglia in naïve retina analyzed with two‐way ANOVA *p* = < 0.0001 (D) Mixed‐effects analysis of acute IOP datasets. Mouse‐level STING abundance is shown across age, mtDNA background, and treatment groups. Linear mixed‐effects modeling identified significant effects of age (*p* = 0.0003), treatment (*p* = 0.0073), and a genotype × age interaction (*p* = 0.0389). (E) Genotype‐specific comparisons derived from the mixed‐effects model. Under control conditions, MNX mice exhibited higher IBA1 STING abundance than WT mice at 2 months (*p* = 0.0246)(*), whereas WT mice exhibited higher IBA1 STING abundance than MNX mice at 20 months (*p* = 0.0455)(*), demonstrating an age‐dependent reversal in genotype‐associated microglial STING abundance. (F) Treatment‐specific comparisons derived from the mixed‐effects model. Acute IOP elevation was associated with reduced microglial STING abundance in 20‐month‐old WT mice relative to contralateral control eyes (*p* = 0.0238). No other treatment contrasts reached statistical significance. Error bars represent 95% confidence intervals.

### 3.5 Age Reduces Lysosomal Colocalization of STING

At 20 months, raw, non‐proportioned, STING levels remained elevated in microglia across both C3H and C57 mtDNA backgrounds, with naïve levels showing STING puncta levels comparable to those observed after acute IOP elevation (see Supporting Information). At 20 months of age there are increased STING levels inside microglia in the naïve eyes, with higher levels of STING in microglia of the normotensive eye than the 50‐mmHg eye. This data is non‐ normalized to the amount of microglia in each image and thus could be biased by this lack of standardization. However, this exploratory observation motivated subsequent analyses examining whether age‐related changes in STING abundance were accompanied by alterations in STING‐LAMP1 spatial association. Lysosomal‐associated membrane protein 1 (LAMP1) and STING analysis by three‐way ANOVA (Figure 6) revealed no significant differences in STING‐LAMP1 colocalization across mtDNA backgrounds (*p* = 0.1909) or in response to acute IOP stress (*p* = 0.504). However, aging was associated significantly (*p* = 0.002) with reduced STING‐LAMP1 colocalization, with 20‐month‐old mice exhibiting lower levels compared to younger groups when evaluated using Tukey's post hoc (20 month vs. 10 month *p* = 0.031, 20 month vs. 2 month *p* = < 0.001) (Figure 6C).

**FIGURE 6:**
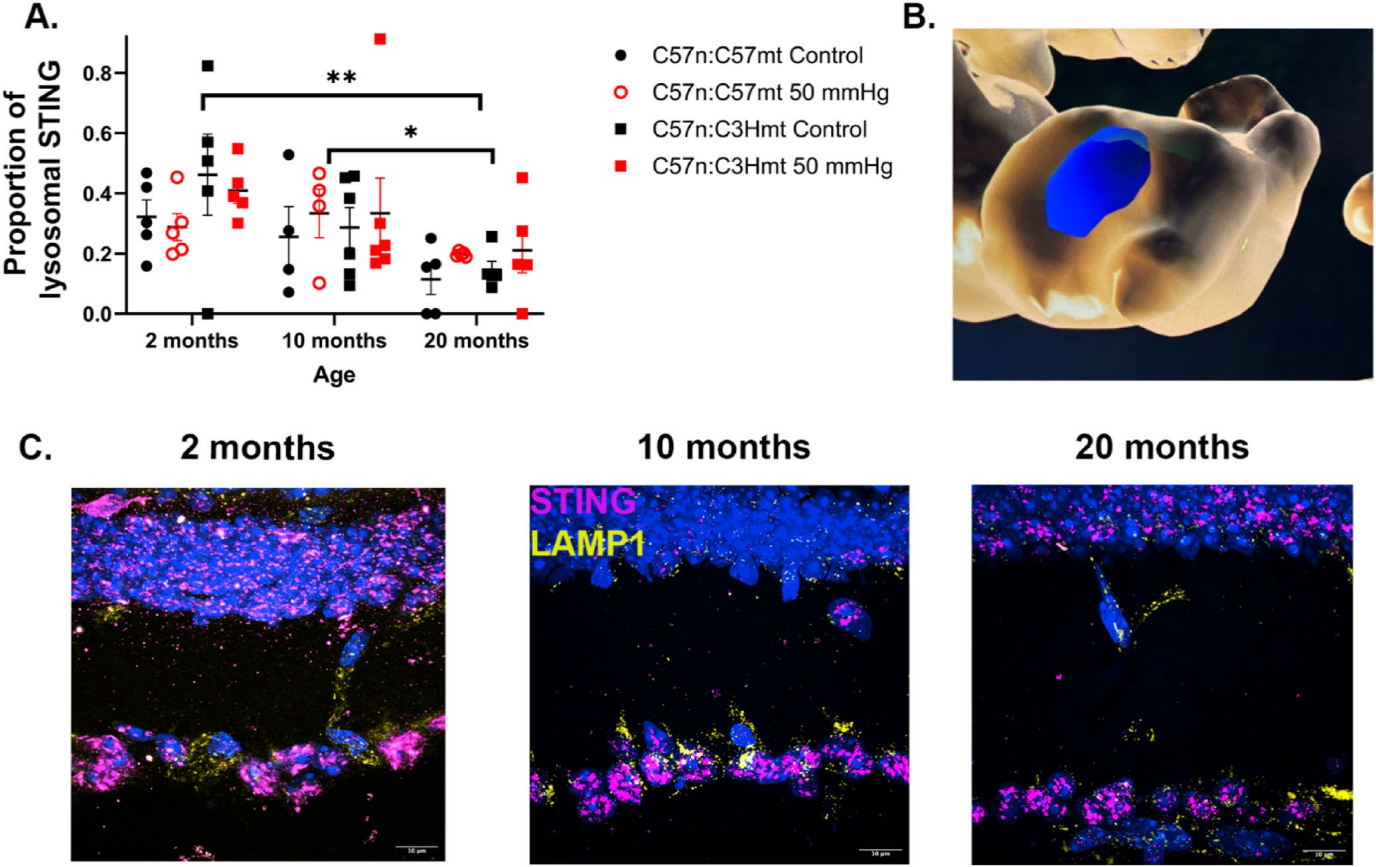
Age results in decline of lysosomal STING, regardless of mtDNA background or acute IOP stress. (A) Original immunohistochemistry, (Magenta, STING, Yellow, LAMP1), scale bar = 20 μm. (B) Schematic image of IMARIS analysis using surface creation, orange, LAMP1, blue, STING. (C) Graph of LAMP1 and STING colocalization; X: Age of mice, Y: Proportion of LAMP1‐STING normalized to total amount of STING, analyzed with Three‐way ANOVA, Tukey's post hoc, *p* = < 0.001(**) (20 months vs. 2 months) and 0.03(*) (10 months vs. 20 months)

## 4 Discussion

Bioenergetics, aging, and immunometabolism are increasingly recognized as interconnected drivers of chronic disease across multiple tissues, including the central nervous system (Płóciniczak et al. 2025; Rahimpour et al. 2025). In cardiovascular and metabolic disease research, this relationship has been explored using mouse models that isolate mtDNA variation (Figure 1A), demonstrating that C57/BL6 mtDNA confers greater susceptibility to pathological stress in cardiac tissue (Betancourt et al. 2014). However, whether this paradigm extends to the retina remains unclear. Age complicates this relationship as a non‐modifiable factor influencing metabolism, function, inflammation, and oxidative stress (Franceschi et al. 2018). Here, we identify associations between mtDNA polymorphisms, metabolic remodeling, and differential inflammatory protein expression in the retina.

The retina provides a uniquely sensitive system to study mitochondrial–immune interactions in the central nervous system. It combines high energy demand (Chen et al. 2024), susceptibility to oxidative and mtDNA damage, tightly coupled neuron–immune interactions, and quantitative functional readouts that are highly responsive to mitochondrial perturbation (Figure 1D). In addition, established acute stress models further enhance its utility for probing how mitochondrial genetics and aging influence bioenergetics, function, and immunometabolism (Figure 1E).

The C57 and C3H mtDNAs differ by 2 missense mutations in NADH dehydrogenase (np 9461 A to C transversion in subunit 3) and cytochrome c oxidase (np 9348 G to A transition in subunit 3), which have been previously associated with bioenergetic differences in liver and heart, respectively (Fetterman et al. 2013). Our data suggest age‐related nuclear compensation in mitochondrial composition (Figure 2), particularly involving complexes III, IV, and V. We observed a pronounced decline in the nuclear encoded complex III subunit UQCRC2 between 2 and 20 months. Because complex III is a major site of reactive oxygen species (ROS) generation via the Q cycle (Cape et al. 2006; Cramer et al. 2011; Zhao et al. 2019), this downregulation may represent a compensatory response to limit oxidative stress, consistent with the observed age‐dependent increase in mtDNA damage, though methods that directly quantify ROS will be needed to substantiate this connection. Given that ROS can signal back to the nucleus to regulate gene expression (Barnett et al. 2025; Darfarin and Pluth 2025), we hypothesize that this reduction reflects mito‐nuclear feedback, although further validation using techniques that directly assess transcriptional changes is needed.

Consistent with prior studies (Daum et al. 2013; Ebanks et al. 2020; Galber et al. 2021; Martín‐Maestro et al. 2019; Orr et al. 2019; Vidali et al. 2023), complex V levels decreased with age in the retina, while complex IV levels increased. This divergent pattern resembles mitochondrial adaptations observed in ATP synthase deficiency (Havlíčková Karbanová et al. 2012), where upregulation of complex IV may preserve electron transport and reduce upstream ROS accumulation when ATP production is compromised. Interestingly, MNX retinas selectively show age‐related changes in complexes I and II, while the levels of these complexes remain stagnant in the WT mice with age, generating an age and mtDNA‐dependent observation. Complexes I and II serve as major entry points for electrons into the electron transport chain, and their MNX‐selective age‐related upregulation could reflect altered metabolic demand or compensatory changes in mitochondrial metabolism. Differential reliance on glycolysis or other metabolic pathways may contribute to this response, although additional experiments would be required to determine the contribution of these pathways. Experiments that further account for changes in mitochondrial abundance, including employing the use of citrate synthase could further sharpen the findings here that are normalized to beta actin, a cytoskeletal protein.

Together, these findings suggest an age‐associated remodeling of OXPHOS composition that may reflect coordinated mitonuclear interactions. Additional studies will be necessary to determine whether these changes are mediated by mitonuclear signaling and to identify the specific mechanisms underlying this remodeling.

Despite significant differences in OXPHOS complex abundance between MNX and WT at 2 months of age, electroretinography at both 3 weeks and 2 months revealed reduced *b*‐wave amplitudes in mice with C57 mtDNA, indicating impaired inner retinal function, primarily reflecting ON bipolar cell function (Bhatt et al. 2023; Snellman et al. 2008). These cells have high ATP demands due to sustained synaptic activity (Heidelberger et al. 2002), suggesting that mtDNA‐dependent differences in mitochondrial performance may be associated with limited function. We hypothesize that the high membrane potential associated with C57 mitochondria (Betancourt et al. 2014) promotes metabolic rigidity, increased ROS production (Esteras Gallego et al. 2015), and reverse electron transport pressure (Bao et al. 2025), impairing the ability to dynamically meet fluctuating energy demands. Alternative explanations, including differences in retinal perfusion or developmental variation between strains, may also contribute to the observed functional differences and warrant further investigation. At this stage, in the absence of ERG data at the 10‐ and 20‐month timepoints, it is unclear how changes in OXPHOS composition alter retinal firing with age.

Additionally, given results of an exploratory analysis of STING‐IBA1 IOP experiments (Supporting Information), we examined the protein expression and intracellular localization of STING to assess changes in response to mtDNA polymorphisms and age. Altered microglial STING dynamics have been shown to be a driver of inflammaging, defined as chronic, low‐grade inflammation that increases with age (Franceschi et al. 2018). Microglia showed an age‐dependent increase in STING abundance, consistent with increased mtDNA damage results (Figure 4). However, whether these alterations are accompanied by functional activation of the STING pathway remains to be determined. Future studies that assess the altered interferon response could provide deeper conclusions on the direct impact of mtDNA polymorphisms on inflammaging. In parallel, lysosomal localization of STING decreased with age, regardless of mtDNA, consistent with altered STING trafficking or lysosomal processing and potentially contributing to STING accumulation in aged microglia (Figure 6). Additional experiments will be required to determine whether altered lysosomal degradation contributes to this phenotype.

Notably, mixed‐effects analysis revealed that microglial STING abundance was significantly influenced by age and acute IOP elevation and exhibited a significant genotype × age interaction. Acute IOP exposure was generally associated with lower STING abundance than contralateral control eyes, with a significant reduction observed in aged WT animals. These findings are consistent with altered regulation of STING abundance following retinal stress; however, the mechanisms underlying this reduction remain unclear. Future studies examining additional components of the STING pathway may help determine whether these changes are associated with altered STING activation, trafficking, degradation, or other regulatory processes. These findings further suggest that mitochondrial genetic background may influence age‐dependent patterns of STING regulation during retinal stress. However, because only total STING abundance was quantified and only a single 1‐h recovery timepoint was examined, the extent to which these changes reflect altered STING pathway activity remains unknown. Future studies incorporating downstream signaling analyses, multiple post‐injury recovery intervals, and chronic IOP paradigms will be necessary to determine the functional consequences of these observations and to understand how mitochondrial genetic background and aging influence long‐term retinal stress responses, retinal ganglion cell survival, and neuroprotection.

Our findings support a working model in which mitochondrial stress, aging, and mtDNA variation are associated with coordinated changes in retinal bioenergetics and neuroimmune phenotype. This study demonstrates an age‐related increase in mtDNA lesion burden, with reduced damage in C3H mtDNA at 20 months. Although damaged mtDNA has the potential to engage cGAS‐STING signaling, our study did not directly assess cytosolic mtDNA, cGAS activity, or downstream STING pathway activation. Thus, the observed association between mtDNA lesion burden and STING abundance raises the possibility that mitochondrial stress contributes to age‐associated changes in retinal innate immune regulation, but the mechanistic relationship remains to be determined.

### 4.1 Study Limitations

A limitation of this study is the absence of electroretinographic measurements at the 10‐ and 20‐month timepoints, which precludes direct functional correlation with age‐dependent molecular changes. Longitudinal acquisition of these data would require extended aging of experimental cohorts beyond the scope of the current study. However, the early functional differences observed suggest that mtDNA‐dependent effects on retinal signaling are established prior to advanced aging, and prior studies indicate that mitochondrial‐dependent functional differences may converge at later ages. We also acknowledge that the relatively small sample sizes in several experimental groups limit statistical power and the ability to detect smaller effects, particularly interactions. While we did not observe a consistent sex‐associated pattern in the datasets examined, the limited sample sizes precluded adequately powered statistical evaluation of sex‐specific effects in several molecular datasets. As this study continues and additional biological replicates are compiled, sex‐based differences can be explored in full capacity.

## 5 Conclusion

Mitochondrial genetic diversity is widespread in human populations (Wallace et al. 1995), highlighting the importance of mtDNA variation in shaping bioenergetics, aging, and disease susceptibility, including in the central nervous system. Interactions among bioenergetics, aging, and immunometabolism are critical determinants of tissue stress responses and chronic disease risk. In this study, we demonstrate age‐associated mitochondrial remodeling consistent with mtDNA–nuclear crosstalk and identify mtDNA‐dependent differences that could ultimately indicate changes in neuroinflammatory signaling. Specifically, we show that mtDNA background and aging are associated with increased microglial STING abundance and reduced STING–LAMP1 localization with age, suggesting that mitochondrial genetic variation may contribute to age‐associated differences in retinal immunometabolic homeostasis.

## Disclaimer

All animal studies were conducted in compliance with the National Institutes of Health Guide for the Care and Use of Laboratory Animals and were approved by the Institutional Animal Care and Use Committee of the University of Alabama at Birmingham.

## Author Contributions

Somerville, Ballinger, and Gross conceived the research. Somerville performed experiments except for electroretinograms, which were performed by Lin. Somerville and Lin analyzed and interpreted the data. Somerville and Gross drafted the manuscript, with revisions, edits, and approval from all authors.

## Funding

This work was supported by internal institutional impact funds from the Heersink School of Medicine at the University of Alabama at Birmingham.

## Conflicts of Interest

The authors declare no conflicts of interest.

## Supporting information


**Figure S1:** Age leads to naïve accumulation of STING protein inside of microglia. At 10 months of age, naïve STING levels inside microglia, as analyzed in Imaris software, is negligible. This increases in the contralateral normotensive eye and increases further in eye that has undergone an acute IOP increase (50 mmHg). At 20 months of age there are increased STING levels inside microglia in the naïve eyes, with higher levels of STING in microglia of the normotensive eye than the 50 mmHg eye. These data is non‐normalized to the amount of microglia in a given image and thus could be biased by this lack of standardization. However, this observation serves as the basis for the exploration of how potential age‐related differences in STING could be from lack of STING degradation.
**Data S1:** Mixed‐effects analysis of microglial STING abundance following acute IOP elevation. Acute IOP datasets were analyzed using a linear mixed‐effects model with age, mtDNA background, treatment (control or 50 mmHg IOP), and their interactions as fixed effects and MouseID as a random intercept to account for paired contralateral eyes. Post hoc pairwise comparisons were performed using estimated marginal means (emmeans) with Tukey adjustment.

## Data Availability

The datasets generated and analyzed during the current study, excluding spreadsheets corresponding to IMARIS image and electroretinogram analyses, are available from the corresponding author upon reasonable request. IMARIS image analysis and electroretinogram datasets are available via Dryad. Supporting data are also included within the article and its Supporting Information.
