# Supplementary material for: Mitochondrial Genetic Background Shapes Baseline Retinal Function, Age‐Related Bioenergetic Remodeling, and Microglial STING Phenotypes
Source: Aging Cell. 2026 Oct 15;25(10):e70771. doi: 10.1111/acel.70771 (PMC13670412; doi:10.1111/acel.70771)
Supplement: Supplementary file 1 [file ACEL-25-e70771-s001.docx]

A.

B.

**Supporting Information figure 1. Age leads to naïve accumulation of STING protein inside of microglia.** At 10 months of age, naïve STING levels inside microglia, as analyzed in Imaris software, is negligible. This increases in the contralateral normotensive eye and increases further in eye that has undergone an acute IOP increase (50 mmHg). At 20 months of age there are increased STING levels inside microglia in the naïve eyes, with higher levels of STING in microglia of the normotensive eye than the 50 mmHg eye. This data is non-normalized to the amount of microglia in a given image and thus could be biased by this lack of standardization. However, this observation serves as the basis for the exploration of how potential age-related differences in STING could be from lack of STING degradation.

| Analysis | Description | Results |
| --- | --- | --- |
| Overall mixed-effects model | Linear mixed-effects model: mean STING abundance ~ Age × Genotype (mtDNA) × Treatment, with MouseID included as a random intercept to account for paired contralateral eyes. | Type III Analysis of Variance Table with Satterthwaite's method  Sum Sq Mean Sq NumDF DenDF F value Pr(>F)  Genotype 0.004700 0.004700 1 28.144 0.6917 0.4125881  Treatment 0.058386 0.058386 1 23.856 8.5930 0.0073285  Age 0.160296 0.080148 2 23.619 11.7958 0.0002805  Genotype:Treatment 0.001117 0.001117 1 23.856 0.1644 0.6887371  Genotype:Age 0.049600 0.024800 2 28.307 3.6499 0.0388818  Treatment:Age 0.003255 0.001627 2 23.846 0.2395 0.7888749  Genotype:Treatment:Age 0.024851 0.012426 2 23.846 1.8287 0.1824333 |
| Age contrast | Estimated marginal means (emmeans) pairwise comparisons of age within each genotype (mtDNA) × treatment group, with Tukey adjustment for multiple comparisons. | $contrasts  Genotype = MNX, Treatment = 50mmHg:  contrast estimate SE df t.ratio p.value  10mo - 20mo 0.2019 0.0739 38.3 2.734 0.0250  10mo - 2mo 0.2590 0.0733 41.3 3.531 0.0029  20mo - 2mo 0.0570 0.0733 41.3 0.778 0.7187  Genotype = WT, Treatment = 50mmHg:  contrast estimate SE df t.ratio p.value  10mo - 20mo 0.0216 0.0739 38.3 0.293 0.9540  10mo - 2mo 0.1996 0.0733 41.3 2.722 0.0251  20mo - 2mo 0.1780 0.0733 41.3 2.427 0.0504  Genotype = MNX, Treatment = Control:  contrast estimate SE df t.ratio p.value  10mo - 20mo 0.1813 0.0739 38.3 2.454 0.0482  10mo - 2mo 0.1614 0.0723 43.1 2.232 0.0772  20mo - 2mo -0.0199 0.0723 43.1 -0.275 0.9592  Genotype = WT, Treatment = Control:  contrast estimate SE df t.ratio p.value  10mo - 20mo -0.0299 0.0739 38.3 -0.405 0.9138  10mo - 2mo 0.2598 0.0733 41.3 3.543 0.0028  20mo - 2mo 0.2897 0.0733 41.3 3.950 0.0009 |
| Treatment contrast | Estimated marginal means (emmeans) pairwise comparisons of treatment (Control vs. 50 mmHg IOP) within each age × genotype (mtDNA) subgroup. | $contrasts  Age = 10mo, Genotype = MNX:  contrast estimate SE df t.ratio p.value  50mmHg - Control -0.0148 0.0521 23.6 -0.283 0.7794  Age = 20mo, Genotype = MNX:  contrast estimate SE df t.ratio p.value  50mmHg - Control -0.0354 0.0521 23.6 -0.679 0.5037  Age = 2mo, Genotype = MNX:  contrast estimate SE df t.ratio p.value  50mmHg - Control -0.1123 0.0548 26.9 -2.050 0.0503  Age = 10mo, Genotype = WT:  contrast estimate SE df t.ratio p.value  50mmHg - Control -0.0744 0.0521 23.6 -1.428 0.1664  Age = 20mo, Genotype = WT:  contrast estimate SE df t.ratio p.value  50mmHg - Control -0.1260 0.0521 23.6 -2.416 0.0238  Age = 2mo, Genotype = WT:  contrast estimate SE df t.ratio p.value  50mmHg - Control -0.0143 0.0521 23.6 -0.274 0.7867  Degrees-of-freedom method: kenward-roger |
| Genotype contrast | Estimated marginal means (emmeans) pairwise comparisons of genotype (mtDNA) within each age × treatment subgroup. | $contrasts  Age = 10mo, Treatment = 50mmHg:  contrast estimate SE df t.ratio p.value  MNX - WT 0.1182 0.0739 38.3 1.600 0.1178  Age = 20mo, Treatment = 50mmHg:  contrast estimate SE df t.ratio p.value  MNX - WT -0.0621 0.0739 38.3 -0.841 0.4054  Age = 2mo, Treatment = 50mmHg:  contrast estimate SE df t.ratio p.value  MNX - WT 0.0588 0.0717 47.8 0.820 0.4161  Age = 10mo, Treatment = Control:  contrast estimate SE df t.ratio p.value  MNX - WT 0.0585 0.0739 38.3 0.792 0.4331  Age = 20mo, Treatment = Control:  contrast estimate SE df t.ratio p.value  MNX - WT -0.1527 0.0739 38.3 -2.067 0.0455  Age = 2mo, Treatment = Control:  contrast estimate SE df t.ratio p.value  MNX - WT 0.1569 0.0676 47.1 2.322 0.0246  Degrees-of-freedom method: kenward-roger |

**Supporting Information 2. Mixed-effects analysis of microglial STING abundance following acute IOP elevation.** Acute IOP datasets were analyzed using a linear mixed-effects model with age, mtDNA background, treatment (control or 50 mmHg IOP), and their interactions as fixed effects and MouseID as a random intercept to account for paired contralateral eyes. Post hoc pairwise comparisons were performed using estimated marginal means (emmeans) with Tukey adjustment.
